# Z‑Amino Acids Present Innovative Antimicrobial and Antibiofilm Properties against Methicillin-Susceptible and -Resistant *Staphylococcus aureus*

**DOI:** 10.1021/acsomega.5c09099

**Published:** 2025-12-31

**Authors:** Alexa Sowers, Bingyun Li

**Affiliations:** † Department of Orthopaedics, School of Medicine, 5631 West Virginia University, Morgantown, West Virginia 26506, United States; ‡ Department of Pharmaceutical and Pharmacological Sciences, West Virginia University, Morgantown, West Virginia 26506, United States

## Abstract

One of the challenges associated with bacteria is their ability to form biofilms that can grow on medical equipment, resulting in more severe and persistent infections. Unfortunately, most antibiotics are optimized for planktonic bacteria, and their therapeutic window is limited when targeting biofilms. Eradicating biofilms typically requires concentrations that are much higher than the minimum inhibitory concentration (MIC), increasing the concern about cytotoxicity toward mammalian cells. In this study, benzyloxycarbonyl-protected amino acids (Z-amino acids), for the first time, were analyzed for both antimicrobial and antibiofilm activity against *Staphylococcus aureus* (*S. aureus*), a strain prevalent in surgical infections. It was determined that Z-amino acids, especially Z-glycine (Z-Gly), exhibited fast antimicrobial properties against *S. aureus*, causing early depolarization and leading to enhanced membrane permeability. Severe membrane disruption of *S. aureus* after treatment with Z-glutamine (Z-Gln) was confirmed microscopically. Additionally, Z-amino acids effectively inhibited biofilm formation at 1× MIC and dispersed mature biofilms at 2× MIC. Notably, Z-Gln had the most favorable antimicrobial activity profile with minimal toxicity toward mammalian cells at both 1× and 2× MIC. These concentrations were effective against both planktonic and biofilm-associated bacteria while maintaining at least 80% viability of osteoblast cells after a 24 h exposure. These Z-amino acids also exhibited similar toxicity toward epithelial cells (e.g., BEAS-2B cells), indicating a consistent safety profile against different cell types. These findings suggest that Z-amino acids, particularly Z-Gln, show promise as a new therapeutic agent that targets both planktonic and biofilm-associated infections.

## Graphical abstract

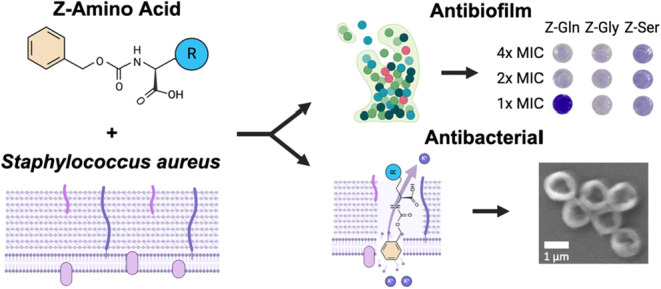

## 1 Introduction

Antimicrobial resistance (AMR) is recognized as an emerging global health crisis of the 21st century.^1,2^ This crisis has become such a problem that AMR is projected to result in approximately two million deaths per year globally.^3^ Although AMR occurs naturally over time due to the ability of microorganisms to adapt and survive in challenging environments, this process is accelerated significantly due to the misuse of antibiotics.^4^ Methicillin-resistant *Staphylococcus aureus* (MRSA) is a prime example of an antibiotic-resistant pathogen that may lead to severe and life-threatening infections.^5^ These bacteria have become problematic in clinical settings, where they can adhere and grow on medical equipment, such as implants and catheters, making patients more vulnerable to serious infections.^6,7^

One of the primary mechanisms associated with AMR is the ability of bacteria to survive on material surfaces through biofilm formation, contributing to persistence and treatment failure. Within a biofilm, there are several factors that contribute to AMR, including the development of an extracellular polymeric substance matrix, which slows the penetration of antimicrobials.^8^ Biofilms may also contain persister cells, which are slow-growing, metabolically inactive cells,^9^ that can evade antimicrobials and allow bacterial regrowth later.^10,11^ Due to their resistance mechanisms, biofilm-forming bacteria may become 10–1000 times more resistant when exposed to antimicrobial agents.^12^ Current antibiotics are typically optimized for planktonic bacteria, often having increased antibiotic minimum inhibitory concentration (MIC) against biofilms,^13^ emphasizing the need to discover novel antimicrobial agents that not only target resistant bacteria but also have the potential to disperse or eradicate biofilms.

To address AMR concerns, recent efforts have been focused on discovering new antimicrobial agents, such as antimicrobial peptides (AMPs). While AMPs have shown strong antimicrobial activity, their development is often limited due to high production costs and batch variations.^14^ Therefore, focusing on amino acids (the building blocks of peptides), amino acid analogues, or amino acids with their amino or carboxyl groups protected (protected amino acids), which have simple structures and are relatively easy to synthesize, offers a promising alternative. Protected amino acids, such as those protected with fluorenylmethyloxycarbonyl (Fmoc) and *tert*-butoxycarbonyl (Boc), are commonly used as precursors for peptide synthesis. However, many of these protected amino acids have not been studied for their independent biological or antimicrobial activity. Previous preliminary studies have focused on the self-assembling and antimicrobial properties of Fmoc- and Boc-amino acids in combination with an antibiotic to lower the IC_50_ value.^15^ Additionally, Fmoc-amino acids have been synthesized into self-assembling hydrogels and shown to present antimicrobial properties.^14,16–22^ Fmoc-phenylalanine, in particular, has shown antibiofilm activity, with the ability to inhibit biofilm formation and eradicate mature biofilms, due to surfactant-like characteristics.^23^ These findings highlight the potential of protected amino acids as antimicrobial agents. D-Amino acids have also been shown to have some antibiofilm activity, primarily through biofilm inhibition,^24–26^ dispersal,^27–29^ and enhancing the activity of antibiotics when used in combination.^30–32^

The benzyloxycarbonyl (Z) group is a widely used protecting group for amines, which is a favorable option due to its easy removal and high stability in most basic and acidic environments.^33^ Compared to other protecting groups such as Fmoc, Z-groups are simpler and less hydrophobic, but they still have hydrophobic groups that can exhibit surfactant-like properties.^18,34^ Although Z-protected amino acids are commercially available and have been used in peptide synthesis, Z-amino acids have not been evaluated for both antimicrobial and antibiofilm activity. Given these properties, Z-amino acids represent a promising unexplored class of small molecules with potential dual-functional activity.

In this study, we determined the antimicrobial and antibiofilm properties of benzyloxycarbonyl (Z)-protected amino acids (Z-amino acids), which can be used as building blocks to synthesize new AMPs with desired properties of interest, against both methicillin-susceptible *S. aureus* (MSSA) and MRSA strains. We hypothesized that Z-amino acids exhibit antimicrobial and antibiofilm properties through membrane permeability mechanisms.

## 2 Materials and Methods

### 2.1 Materials

Unprotected amino acids [i.e., l-glutamine (l-Gln), l-glycine (l-Gly), and l-serine (l-Ser)], Z-protected amino acids [i.e., *N*-benzyloxycarbonyl-l-glutamine (Z-Gln), *N*-benzyloxycarbonyl-l-glycine (Z-Gly), and *N*-benzyloxycarbonyl-l-serine (Z-Ser)], Triton-X, Bis­(1,3-Dibutylbarbituric Acid) Trimethine Oxonol [DiBAC_4_(3)], dimethyl sulfoxide (DMSO), propidium iodide (PI), brain heart infusion (BHI), hexamethyldisilazane (HMDS), d-glucose, crystal violet, and acetic acid were purchased from Millipore Sigma (Burlington, MA, USA). Blood agar (Tryptic Soy Agar with 5% sheep blood) plates were obtained from Remel (Lenexa, KS, USA). Mueller Hinton broth (MHB) was purchased from BD Difco (Sparks, MD, USA), and ethanol was purchased from Decon Laboratories (King of Prussia, PA, USA). Fetal bovine serum (FBS), penicillin-streptomycin solution (100×), Dulbecco’s modified Eagle’s medium (DMEM) containing 1 g/L, l-Gln, and sodium pyruvate, untreated polystyrene 96-well flat-bottom plates, and tissue culture-treated 96-well flat-bottom plates were purchased from Corning (Corning, NY, USA). Glutaraldehyde was purchased from Electron Microscopy Sciences (Hatfield, PA, USA). Methanol was purchased from Fisher Chemical (Fair Lawn, NJ, USA). 0.25% Trypsin-ethylenediaminetetraacetic acid (EDTA) and Dulbecco’s phosphate-buffered saline (PBS) without calcium chloride and magnesium chloride were purchased from Gibco (Grand Island, NY, USA). The CyQUANT MTT cell viability assay kit, 0.2 μm poly­(ether sulfone) (PES) syringe filters, and Minimum Essential Medium α (MEM α) with nucleosides were purchased from Thermo Fisher Scientific (Waltham, MA, USA).

MSSA (ATCC 25923) and MRSA (BAA 1717) were purchased from ATCC (Manassas, VA) and studied. BEAS-2B cells were obtained from the National Institute for Occupational Safety and Health at Morgantown, WV, and rat osteoblast cells were purchased from Cosmo Bio Co (Tokyo, Japan). These cells were maintained according to standard cell culture protocols, similar to our previous studies.^35–37^

### 2.2 Selection of Z-Amino Acids

Z-amino acids are commercially available and are primarily used in peptide synthesis. In this study, Z-amino acids were studied to determine how they influence antimicrobial activity and potential cytotoxicity. Z-Gln, Z-Gly, and Z-Ser were selected based on their diverse properties, including differences in size, side-chain chemistry, and relevant biological characteristics. Z-Gln was selected because Gln has been reported to positively influence processes related to wound healing,^38–40^ making its Z-protected form interesting for potential therapeutic applications in infected wounds. Z-Gly was included because Gly is the smallest amino acid and has a single hydrogen atom as its side chain, allowing for evaluation of the effects of Z-protection without steric interactions. Z-Ser was chosen because Ser is a polar amino acid with a hydroxyl side chain that can form hydrogen bonds, which may enhance solubility. Due to the hydrophobic nature of Z-protecting groups, combining them with a polar residue is important in maintaining solubility at the concentrations tested. Therefore, bulky hydrophobic Z-protected residues, such as Z-phenylalanine, Z-leucine, and Z-tryptophan, were not included in this study due to issues with solubility and aggregation at concentrations required for antimicrobial activity. Additionally, positively charged residues, such as Z-lysine and Z-arginine, were excluded from this study to evaluate whether Z-protection contributed to antimicrobial activity without additional bacterial selectivity due to electrostatic interactions.

### 2.3 Antimicrobial Tests

MSSA and MRSA strains were cultured in BHI broth. Three isolated colonies from a blood agar plate streaked with MSSA or MRSA were inoculated into 20 mL BHI, which were incubated at 37 °C with shaking at 200 revolutions per minute (rpm).^41^ After incubation for 16 h, the inoculum was diluted to 10^7^ colony-forming units/mL (CFU/mL). The inoculum concentration was estimated based on the optical density at 600 nm (OD_600_), which was previously determined by serial dilution and plating. This inoculum was further diluted with fresh BHI broth to obtain a concentration of 10^5^ CFU/mL, which was used for the MIC and minimum bactericidal concentration (MBC) experiments.

Initial stock solutions of 16 mg/mL Z-amino acids (i.e., Z-Gln, Z-Gly, and Z-Ser) and L-amino acids (i.e., l-Gln, l-Gly, and l-Ser) were prepared using PBS at pH 6.5. Each initial stock solution was serially diluted 2-fold in 96-well plates using sterile PBS to achieve concentrations 2× higher than the testing concentrations to be studied. Each serial dilution resulted in a final volume of 75 μL, which was mixed with 75 μL of the diluted bacterial inoculum to reach a final volume of 150 μL per well. The final concentrations tested were 0.5, 1, 2, 4, and 8 mg/mL. After addition of the inoculum, the 96-well plate was incubated at 37 °C for 24 h. The MIC was defined as the lowest concentration of the Z-amino acid tested where the solution was not turbid, indicating inhibition of bacterial growth.^42,43^ To count viable bacterial colonies, each well was serially diluted from 10^–1^ to 10^–11^ with sterile PBS. A drop plate method^41,44,45^ was applied where the blood agar plates were divided into four quadrants, each corresponding to a dilution. For each dilution, a 10 μL bacterial suspension was added to the agar, allowed to dry, inverted, and incubated for 24 h at 37 °C.^41^ The MBC was determined by plating the bacterial suspension, where the absence of colonies indicated the sample was bactericidal. Triplicate samples were prepared for both MIC and MBC determinations, with the CFU/mL calculated based on counting the colonies from the dilutions.

### 2.4 Time-Inhibition Kinetics

The time-inhibition kinetics of MSSA and MRSA in response to Z-amino acids were studied according to a method from our previous study.^46^ The kinetics experiments were conducted by combining 75 μL of an *S. aureus* suspension (10^5^ CFU/mL) with 75 μL of the Z-amino acid solutions, achieving a final concentration equivalent to the MIC. The treated samples and control (without the addition of Z-amino acids) were incubated for 15, 30, 60, 120, 240, and 360 min at 37 °C while being shaken (200 rpm). After each time point, the samples and control were diluted to 10^–2^, 10^–3^, and 10^–4^ in PBS and tested using the drop plate method described above. Samples were run in triplicate, where the CFU was determined and percent bacterial inhibition was calculated.

### 2.5 Crystal Violet Biofilm Inhibition/Dispersion Assay

The biofilm inhibition assay was modified from an established protocol.^47^ All solutions used in the assays were sterilized by filtration through a 0.2 μm PES filter. The initial stock solutions of Z-amino acids were used, final concentrations of 0.5×, 1×, and 2× MIC were obtained, and each concentration was tested in triplicate. Uninoculated BHI was used as a blank control for crystal violet staining. The untreated polystyrene 96-well plate, containing the experimental and control conditions with the inoculum, was incubated under static conditions for 24 h at 37 °C. The medium in the overnight culture containing either Z-amino acids or control medium was removed from each well by aspiration. The wells were then washed three times with sterile PBS to remove nonadherent bacteria. After washing, the adherent bacteria were treated with 100 μL of 99% (v/v) methanol for 15 min. Any remaining methanol was allowed to evaporate in a laminar flow hood. The biofilms were then stained with 0.5% crystal violet for 5 min. Any excess crystal violet was washed with deionized water three times to remove unbound stain. The stain bound to the biofilms were solubilized with 200 μL of 33% acetic acid, and the absorbance was measured at 570 nm using a microplate reader. The biofilm dispersion assay was conducted similarly to the biofilm inhibition assay, but the inoculum was allowed to form mature biofilms for 48 h in untreated polystyrene 96-well plates before any treatment.^48^ L-Amino acids were included as controls in antimicrobial and biofilm inhibition assays. Since no significant antimicrobial properties were observed, L-amino acids were not evaluated further in the biofilm dispersion or cell viability assays.

### 2.6 Bacterial Membrane Permeabilization and Depolarization

Membrane permeabilization and depolarization experiments were conducted^46,49^ to determine the antimicrobial mechanism. For the determination of membrane permeabilization, *S. aureus* (MSSA) was incubated for 18 h at 37 °C while being shaken (200 rpm). After incubation, 100 μL of the culture was diluted with 9.9 mL of fresh MHB and incubated for 2 h. The culture was centrifuged at 4000*g* for 7 min and washed twice with PBS supplemented with 25 mM glucose. The culture was then adjusted to 1 × 10^7^ CFU/mL using PBS with 25 mM glucose. Solutions of Z-Gln, Z-Gly, and Z-Ser were prepared at 4× MIC, where Triton-X was used as a positive control. A stock solution of 20 mM PI was prepared in the dark using DMSO, which was further diluted to obtain 1 mM PI with deionized water. For the assay, 25 μL of the Z-amino acid, 2 μL of PI (1 mM), and 73 μL of *S. aureus* (total of 100 μL) were added to a black 96-well plate and incubated at 37 °C with shaking (200 rpm). The samples were read at 15, 30, 60, 120, 240, and 360 min at excitation and emission wavelengths of 584 and 620 nm, respectively. For the depolarization experiments, DiBAC_4_(3) was prepared at 10 μM. Then, 5 μL of DiBAC_4_(3) and 70 μL of *S. aureus* were added to the well and incubated for 15 min. After incubation, 25 μL of the 4× MIC Z-amino acid (total of 100 μL) was added to a black 96-well polystyrene plate and incubated at 37 °C with shaking (200 rpm). The samples were read at excitation and emission wavelengths of 485 and 520 nm, respectively, and fluorescence readings were obtained every 5 min up to 60 min.

### 2.7 Scanning Electron Microscope Analysis

*S. aureus* (MSSA) inoculum was prepared using the same method as in the antimicrobial tests, but the inoculum remained at 10^7^ CFU/mL. The *S. aureus* morphology was assessed using a scanning electron microscope (SEM) with a modified protocol.^50^ Morphological changes at 0.5× and 1× MIC were determined by diluting an initial stock (16 mg/mL) to each concentration with a final volume of 1 mL, including the bacterial inoculum. A control was prepared by combining the buffer with the inoculum. After vortexing, the samples were incubated at 37 °C for 5 h with shaking at 200 rpm. After incubation, the treatments and control were centrifuged at 4000*g* for 5 min. The supernatant was removed, and the pellet was washed with 1 mL of PBS. This process was repeated once to obtain the final pellet for SEM determination. The pellet was used to make a smear on a glass slide using a pipet tip, and the smear was covered with 2 mL of 2.5% glutaraldehyde and left for 1 h at room temperature for fixation. The smear was then washed with PBS followed by a graded series of ethanol (30%, 50%, 70%, 80%, 90%, and twice at 100%) at room temperature, with each step lasting 10 min. Next, the slides were chemically dried using HMDS. The slides were gold sputtered using the Denton Desk V Sputter Coater and analyzed using SEM (JEOL JSM-7600F).

### 2.8 Cell Viability

Rat osteoblast cells were suspended in MEM α, and BEAS-2B cells were suspended in DMEM. Adherent cells were detached from flasks using 0.25% trypsin-EDTA prior to seeding. Both cell types were seeded at a density of 1.0 × 10^4^ cells/well in a 96-well plate and incubated for 24 h to allow for adherence. Both cell types were exposed to 100 μL of Z-amino acids at concentrations of 0.5, 1, 2, and 4× MIC for 1, 4, 6, and 24 h with three replicates per treatment. A control group was treated with 100 μL of MEM α or DMEM without Z-amino acids under the same conditions. After treatment with the Z-amino acids, the media were removed and replaced with 100 μL of fresh cell culture medium without phenol-red. The MTT cell viability assay was conducted according to the CyQUANT MTT kit instructions, using MTT reagent and sodium dodecyl sulfate-hydrochloric acid (SDS-HCl) as a solubilizing agent. After the cells were incubated with the solubilizing agent, the absorbance of each well was measured at 570 nm. The blank wells containing only media, MTT, and SDS-HCl (no cells) were subtracted from the absorbance readings for each well. The experiments were repeated twice. Cell viability was calculated by dividing the absorbance of the cells treated with Z-amino acids by the absorbance of control cells containing only media.

### 2.9 Statistical Analysis

Data are presented as mean ± standard deviation, and all statistical analyses were performed using JMP-V18 software. Comparison between two groups was conducted using an unpaired two-tailed *t*-test. For comparisons of more than two groups, raw values were log-transformed to meet the assumptions of normality and equal variance prior to performing a one-way analysis of variance (ANOVA) with Tukey’s honestly significant difference test. Differences were considered statistically significant at *p* < 0.05.

## 3 Results

### 3.1 Antimicrobial Activity of Z-Amino Acids against Planktonic MSSA and MRSA

Z-amino acids exhibited antimicrobial activity against both MSSA and MRSA (Table 1 and Figure 1). The MICs of Z-Gln, Z-Gly, and Z-Ser against MSSA and MRSA were 4 mg/mL (1× MIC). The MBC against MSSA was 8 mg/mL for Z-Gln and 4 mg/mL for both Z-Gly and Z-Ser, while against MRSA it was 8 mg/mL for both Z-Gln and Z-Ser and 4 mg/mL for Z-Gly. All L-amino acid counterparts (l-Gln, l-Gly, and l-Ser) did not exhibit measurable antimicrobial activities at the concentrations tested for both MSSA and MRSA (Table 1).

**Figure 1:**
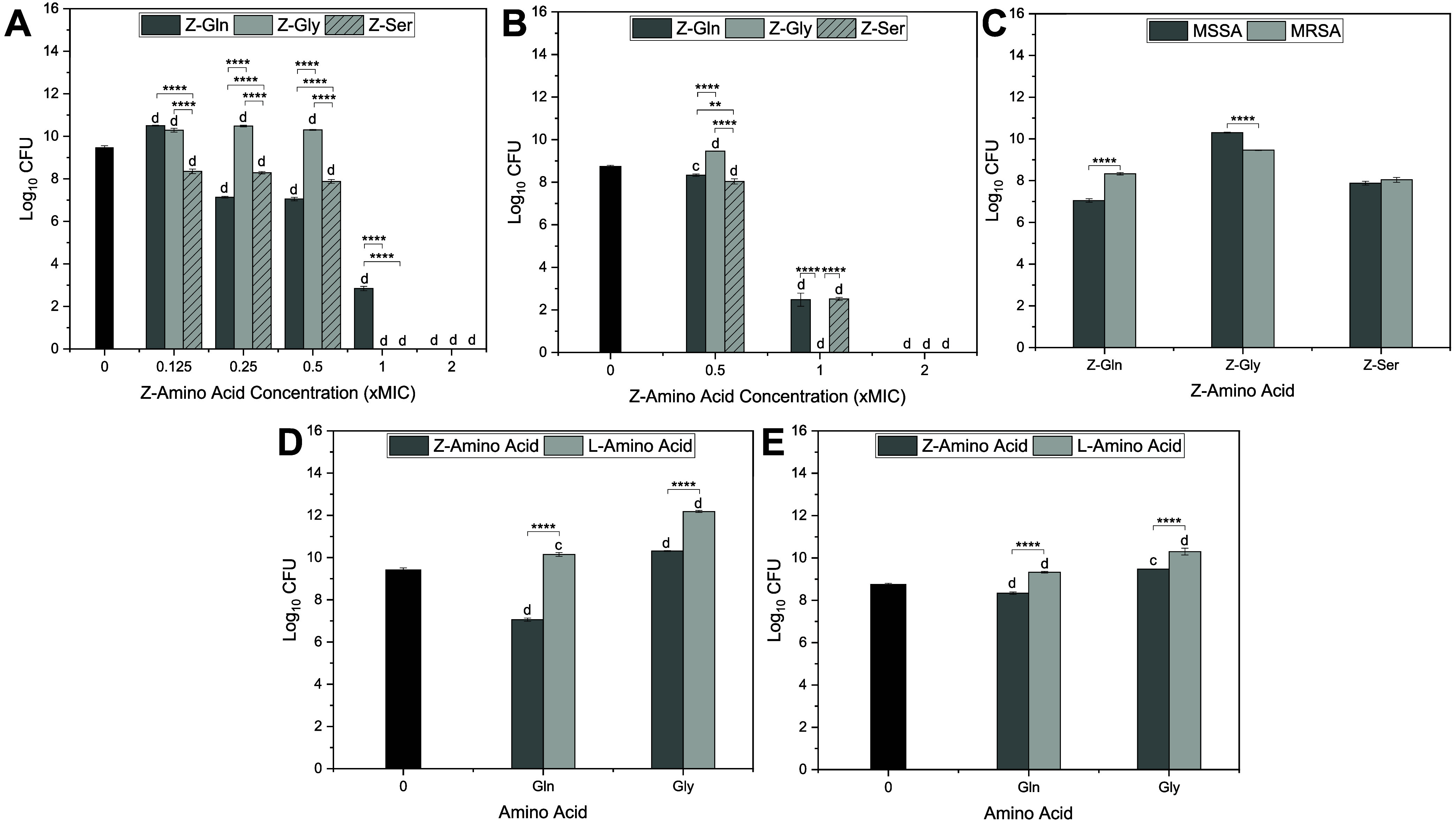
Log_10_ CFU of planktonic *S. aureus* following treatment with Z- and L-amino acids. (A) MSSA and (B) MRSA treated with Z-amino acids at various concentrations. (C) MSSA and MRSA treated with Z-Gln, Z-Gly, and Z-Ser at 0.5× MIC. (D) MSSA and (E) MRSA treated with Z-Gln, l-Gln, Z-Gly, and l-Gly at 0.5× MIC. Statistical significance: ** < 0.01 and **** < 0.0001 compared to the other Z-amino acids (A, B), MSSA vs MRSA (C), and their corresponding L-amino acids (D, E); ^c^ < 0.001 and ^d^ < 0.0001 compared to the control. Data are represented as the mean ± standard deviation with *n* = 3 replicates.

**Table 1:** MIC and MBC of Z- and L-Amino Acids against MSSA and MRSA

MSSA						
	Z-Gln	l-Gln	Z-Gly	l-Gly	Z-Ser	l-Ser
MIC	4 mg/mL	>8 mg/mL	4 mg/mL	>8 mg/mL	4 mg/mL	>8 mg/mL
MBC	8 mg/mL	>8 mg/mL	4 mg/mL	>8 mg/mL	4 mg/mL	>8 mg/mL

MRSA						
	Z-Gln	l-Gln	Z-Gly	l-Gly	Z-Ser	l-Ser
MIC	4 mg/mL	>8 mg/mL	4 mg/mL	>8 mg/mL	4 mg/mL	>8 mg/mL
MBC	8 mg/mL	>8 mg/mL	4 mg/mL	>8 mg/mL	8 mg/mL	>8 mg/mL

Against MSSA (Figure 1A), significant bacterial growth was observed at a low concentration (0.125× MIC) for Z-Gln. Additionally, Z-Gln significantly reduced CFUs compared to the control, 0.25× MIC and above, with no bacterial growth observed at 2× MIC. Compared to the control, Z-Gly significantly increased CFUs at 0.125×, 0.25×, and 0.5× MIC with no bacterial growth observed at 1× and 2× MIC. Z-Ser significantly lowered CFU at all concentrations studied compared to the control and had no bacterial growth at 1× and 2× MIC. At 0.125× MIC, Z-Ser significantly lowered CFUs compared to Z-Gln and Z-Gly. At 0.25× and 0.5× MIC, Z-Gln significantly lowered CFUs compared to Z-Gly and Z-Ser. Z-Ser also had significantly lower CFUs compared to Z-Gly at 0.125×, 0.25×, and 0.5× MIC. At 1× MIC, Z-Gln had significantly higher CFUs compared to Z-Gly and Z-Ser.

Against MRSA (Figure 1B), Z-Gln and Z-Ser significantly reduced CFUs at all concentrations tested, especially at 1× and 2× MIC, when compared to the control. Z-Gly had significant reductions in CFUs at 1× and 2× MIC compared to the control, while it significantly increased CFUs at 0.5× MIC compared to the control. At 0.5× MIC, Z-Gln and Z-Ser significantly reduced CFUs compared to Z-Gly, with Z-Ser showing an additional reduction compared to Z-Gln. At 1× MIC, all Z-amino acids exhibited significantly lower CFUs compared to the control; however, Z-Gly showed an additional reduction compared to Z-Gln and Z-Ser with no bacterial growth. At 2× MIC, all Z-amino acids showed no bacterial growth and presented significant CFU reductions compared to the control.

The treatment effects of Z-amino acids against MRSA and MSSA were compared. It was found that, at 0.5× MIC, MRSA treated with Z-Gln resulted in significantly higher CFU counts than MSSA, while MRSA treated with Z-Gly had a significant reduction in CFUs compared to MSSA. No significant differences were observed between the two strains when treated with Z-Ser (Figure 1C).

The treatment effects of Z-amino acids and L-amino acids against MRSA and MSSA were also compared and assessed at 0.5× MIC. When comparing Z-amino acids to their corresponding L-amino acids against MSSA or MRSA, Z-Gln and Z-Gly resulted in significantly lower CFUs (Figure 1D,E). When compared to the control, L-amino acids (i.e., l-Gln and l-Gly) led to significantly higher CFU against MRSA and MSSA (Figure 1D,E).

The bacterial inhibition kinetics of the Z-amino acids against both MSSA and MRSA were investigated at a concentration of 1× MIC (Figure 2). Against MSSA, Z-Gln inhibited 80% of bacterial growth at 4 h and reached about 100% by 6 h. Z-Ser had similar inhibition kinetics as Z-Gln. Z-Gly demonstrated more rapid activity, inhibiting 90% of bacterial growth within 1 h, and obtained 100% inhibition at 6 h (Figure 2A). Against MRSA, Z-Gly and Z-Ser had similar inhibition kinetics and faster kinetics compared with Z-Gln, with all of them reaching about 100% inhibition at 4 h (Figure 2B).

**Figure 2:**
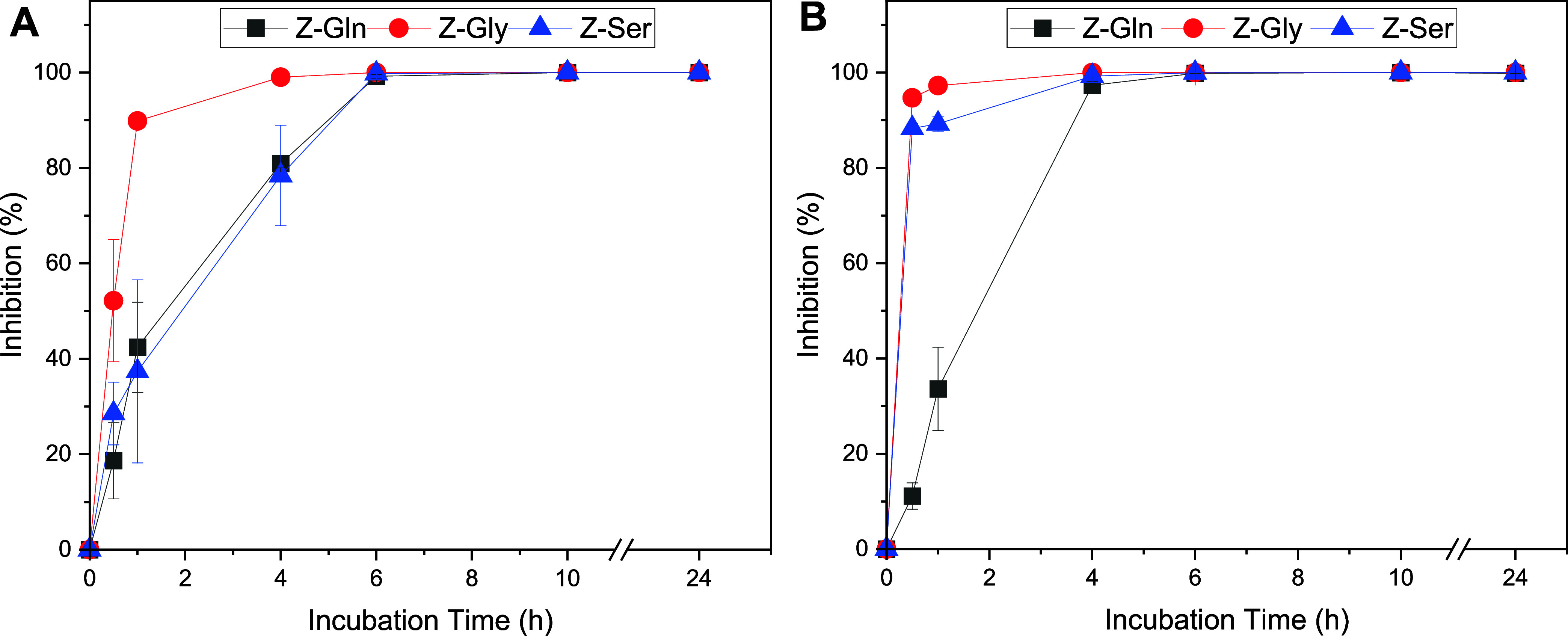
Bacterial inhibition kinetics of *S. aureus* after treatment with Z-amino acids at 1× MIC. Percent inhibition of (A) MSSA and (B) MRSA. Data are represented as the mean ± standard deviation with *n* = 3 replicates.

### 3.2 Biofilm Inhibition and Dispersal Activity of Z-Amino Acids against MSSA and MRSA

Z-amino acids exhibited biofilm inhibition against both MSSA and MRSA, as determined by a crystal violet assay (Figure 3). The relative biofilm biomass was found to be Z-amino acid concentration-dependent. Against MSSA (Figure 3A), Z-Gln and Z-Ser exhibited significantly higher biofilm biomass at 0.5× MIC, increasing by 126% and 268%, respectively, compared to the control. Z-Gly reduced biofilm biomass by 66% at 0.5× MIC, which is significantly lower than the control. Additionally, Z-Gly showed significantly lower biofilm biomass compared to Z-Gln and Z-Ser at 0.5× MIC. Z-Gln reduced biofilm biomass by 85% and 87%, Z-Gly by 82% and 77%, and Z-Ser by 83% and 84% at 1× and 2× MIC, respectively, which were significantly reduced compared to the control. At 2× MIC, Z-Gly had significantly increased biofilm biomass compared to Z-Gln and Z-Ser. Against MRSA (Figure 3B), Z-Gln exhibited no significant reduction in biofilm biomass compared to the control at 0.5× MIC. However, Z-Gly significantly reduced biomass by 41%, whereas Z-Ser significantly increased biomass by 90%, compared to the control at 0.5× MIC. In addition, Z-Gly significantly lowered biofilm biomass compared to Z-Gln and Z-Ser, while Z-Gln significantly reduced biofilm biomass compared to Z-Ser at 0.5× MIC. At 1× MIC, Z-Gln and Z-Ser reduced biofilm biomass by 88%, and Z-Gly reduced biofilm biomass by 89%, with all reductions significant compared to the control. At 2× MIC, Z-Gln and Z-Ser reduced biofilm biomass by 89%, and Z-Gly by 87%, with all reductions being significantly different from the control. At 0.5× MIC, Z-Gln and Z-Ser significantly increased biofilm biomass, whereas Z-Gly significantly reduced biofilm biomass against MSSA compared to MRSA (Figure 3C). Comparing the treatment of Z-amino acids and L-amino acids against MSSA at 1× MIC, Z-Gln and Z-Gly significantly reduced biofilm biomass compared with their corresponding L-amino acids (Figure 3D). No significant differences in the biofilm biomass were observed between the L-amino acid treatments and the control (Figure 3D).

**Figure 3:**
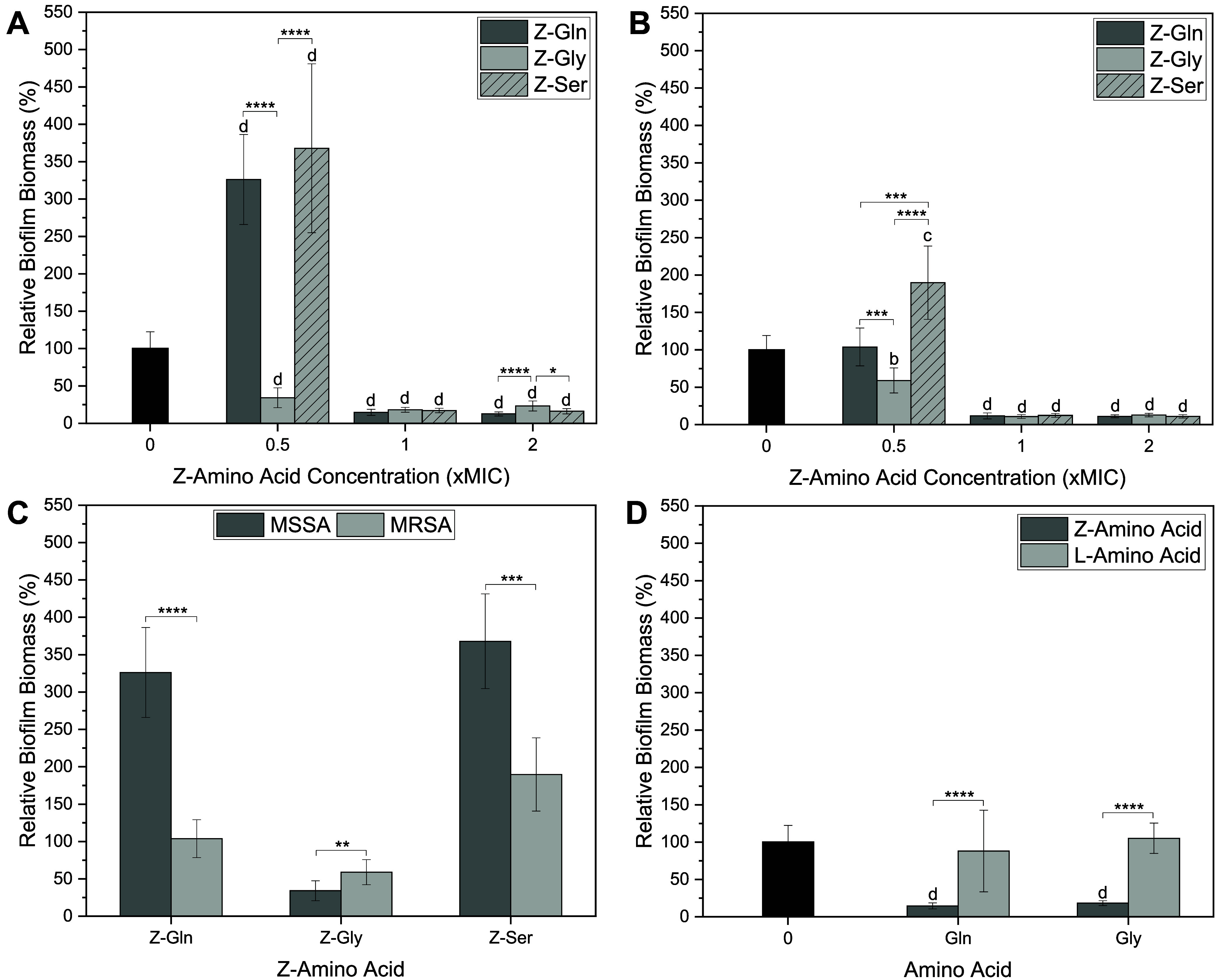
Relative percent of biofilm biomass of (A) MSSA and (B) MRSA treated with Z-amino acids at various concentrations. Comparison in relative percent of biofilm biomass (C) between MSSA and MRSA treated with Z-amino acids at 0.5× MIC and (D) between the treatment of Z-amino acids and L-amino acids against MSSA at 1× MIC. Statistical significance: * < 0.05, ** < 0.01, *** < 0.001, **** < 0.0001 compared to the other Z-amino acids (A–C) or their corresponding L-amino acids (D); ^b^ < 0.01, ^c^ < 0.001, and ^d^ < 0.0001 compared to the control (A, B, D). Data are represented as the mean ± standard deviation with *n* = 6 replicates.

Z-amino acids were also evaluated for their ability to disperse mature biofilms (48 h) of MSSA and MRSA using a crystal violet assay (Figure 4). Against MSSA, Z-Gln exhibited significantly increased biofilm biomass by 47% at 0.5× MIC and 216% at 1× MIC but significantly reduced biofilm biomass by 60% at 2× and 65% at 4× MIC compared to the control. Z-Gly showed no significant difference at 0.5× MIC and significantly reduced biofilm biomass by 70%, 69%, and 52% at 1×, 2× and 4× MIC, respectively, compared to the control. Z-Ser significantly increased biofilm biomass by 51% at 0.5× MIC, showed no significant difference at 1× MIC, and significantly reduced biofilm biomass by 72% and 63% at 2× and 4× MIC, respectively, compared to the control (Figure 4A). At 2× and 4× MIC, all Z-amino acids tested significantly dispersed biofilms compared to the control (Figure 4A), which was visually confirmed by images showing reduced staining (Figure 4B). At 0.5× and 1× MIC, Z-Gly significantly reduced biofilm biomass compared to Z-Gln and Z-Ser; Z-Ser also significantly lowered biofilm biomass compared to Z-Gln at 1× MIC (Figure 4A). Against MRSA, Z-Gln showed no significant difference at 0.5× MIC, but significantly increased biofilm biomass by 425% at 1× MIC compared to the control. At higher concentrations, Z-Gln significantly reduced biofilm biomass by 70% and 65% at 2× and 4× MIC, respectively, compared to the control. For all concentrations tested, Z-Gly significantly reduced biofilm biomass by 54%, 66%, 63%, and 64% at 0.5×, 1×, 2×, and 4× MIC, respectively, compared to the control. Z-Ser significantly increased biofilm biomass by 55% at 0.5× MIC, and significantly reduced biofilm biomass by 73%, 65%, and 67% at 1×, 2×, and 4× MIC, respectively, compared to the control (Figure 4C). Similar to MSSA, at 2× and 4× MIC, all Z-amino acids studied effectively dispersed MRSA biofilms compared to the control (Figure 4C), which was visually confirmed by images (Figure 4D). Moreover, at 0.5× MIC, Z-Gly significantly reduced biofilm biomass compared to both Z-Gln and Z-Ser, with Z-Gln showing a significant reduction in biofilm biomass compared to Z-Ser. At 1× MIC, Z-Gln significantly increased biofilm biomass compared to Z-Gly and Z-Ser (Figure 4C).

**Figure 4:**
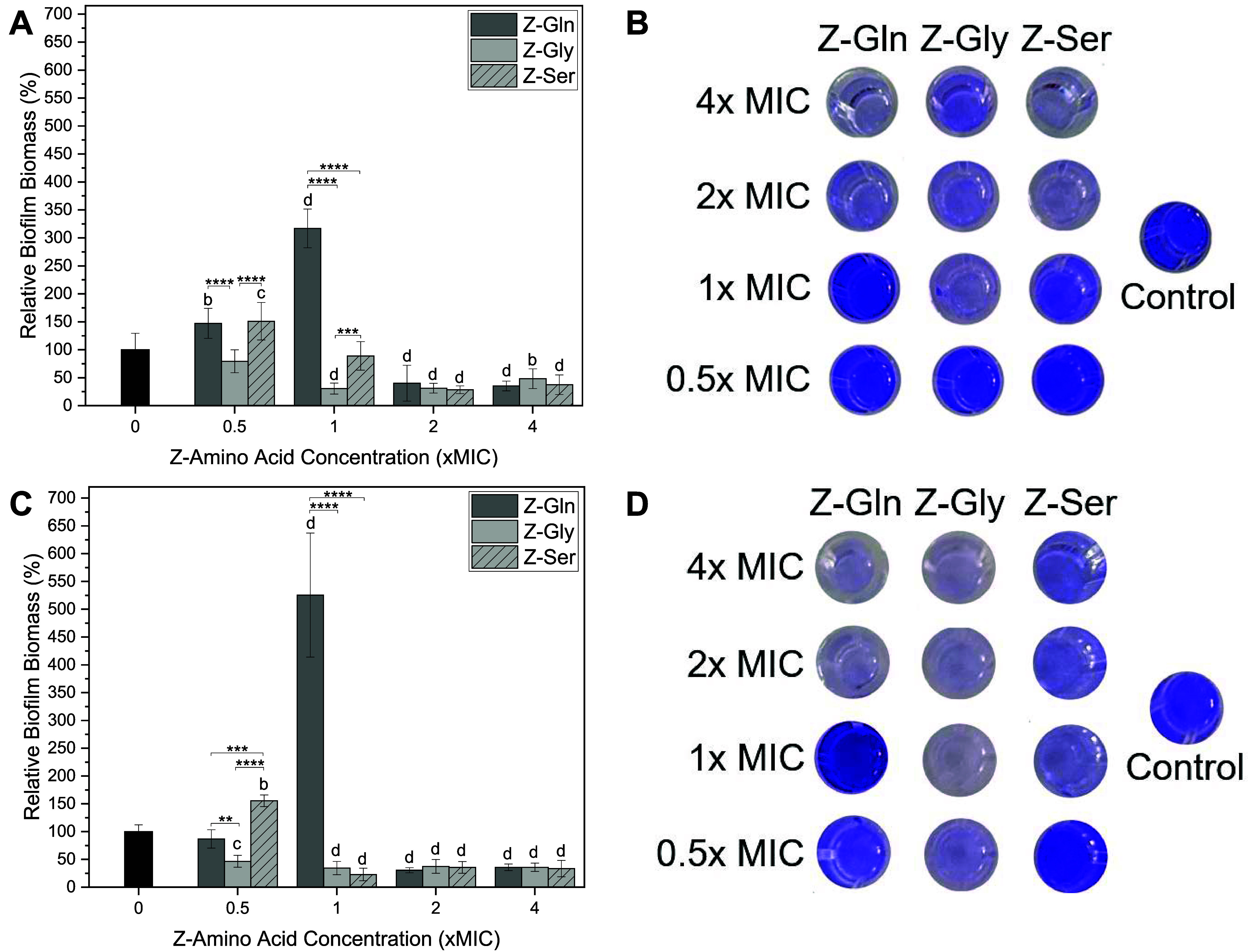
Relative percent of biofilm biomass of (A) MSSA biofilm dispersal after treatment with Z-Gln, Z-Gly, and Z-Ser and (B) representative images of wells stained with crystal violet. (C) MRSA biofilm dispersal after treatment with Z-Gln, Z-Gly, and Z-Ser. (D) Representative images of wells stained with crystal violet. Statistical significance: *** < 0.001, **** < 0.0001; ^b^ < 0.01, ^c^ < 0.001, and ^d^ < 0.0001 compared to the control. Data are represented as the mean ± standard deviation with *n* = 6 replicates.

### 3.3 Antimicrobial Mechanisms of Z-Amino Acids

To gain a better understanding of the antimicrobial mechanism of the Z-amino acids, morphological changes of *S. aureus* and membrane disruption were assessed following exposure to Z-amino acids (Figure 5). Untreated *S. aureus* (Figure 5A) and cells treated with l-Gln (Figure 5B) appeared smooth and spherical, indicating intact cell membranes with no observable damage. Exposure to 0.5× MIC of Z-Gln demonstrated early signs of membrane damage to the cells, including cell deformation and dimpling (Figure 5C). At 1× MIC, Z-Gln treatment led to severe membrane disruption, with cells appearing fully collapsed (Figure 5D). Consistent with the SEM observation, Z-Gln showed significant membrane permeability at 360 min compared to the untreated control (Figure 5E), and exhibited significantly higher membrane depolarization, indicated by higher fluorescence intensity, compared to the untreated control at all time points (Figure 5F). Membrane permeability after exposure to Z-Gly was evident as early as 15 min, with significant uptake of PI compared to the control, and this effect continued for 360 min (Figure 5E). Similar to Z-Gln, Z-Ser exhibited a delayed effect, with significantly higher membrane permeability at 240 and 360 min compared to the untreated control (Figure 5E). The positive control, Triton-X, showed the highest membrane permeability at 240 and 360 min. Membrane depolarization after exposure to Z-Gly and Z-Ser was rapid, with significant increases in fluorescence intensity at all time points tested compared to those of the untreated control (Figure 5F). Triton-X showed significantly higher fluorescence intensity at all time points tested compared to any of the other treatments or control.

**Figure 5:**
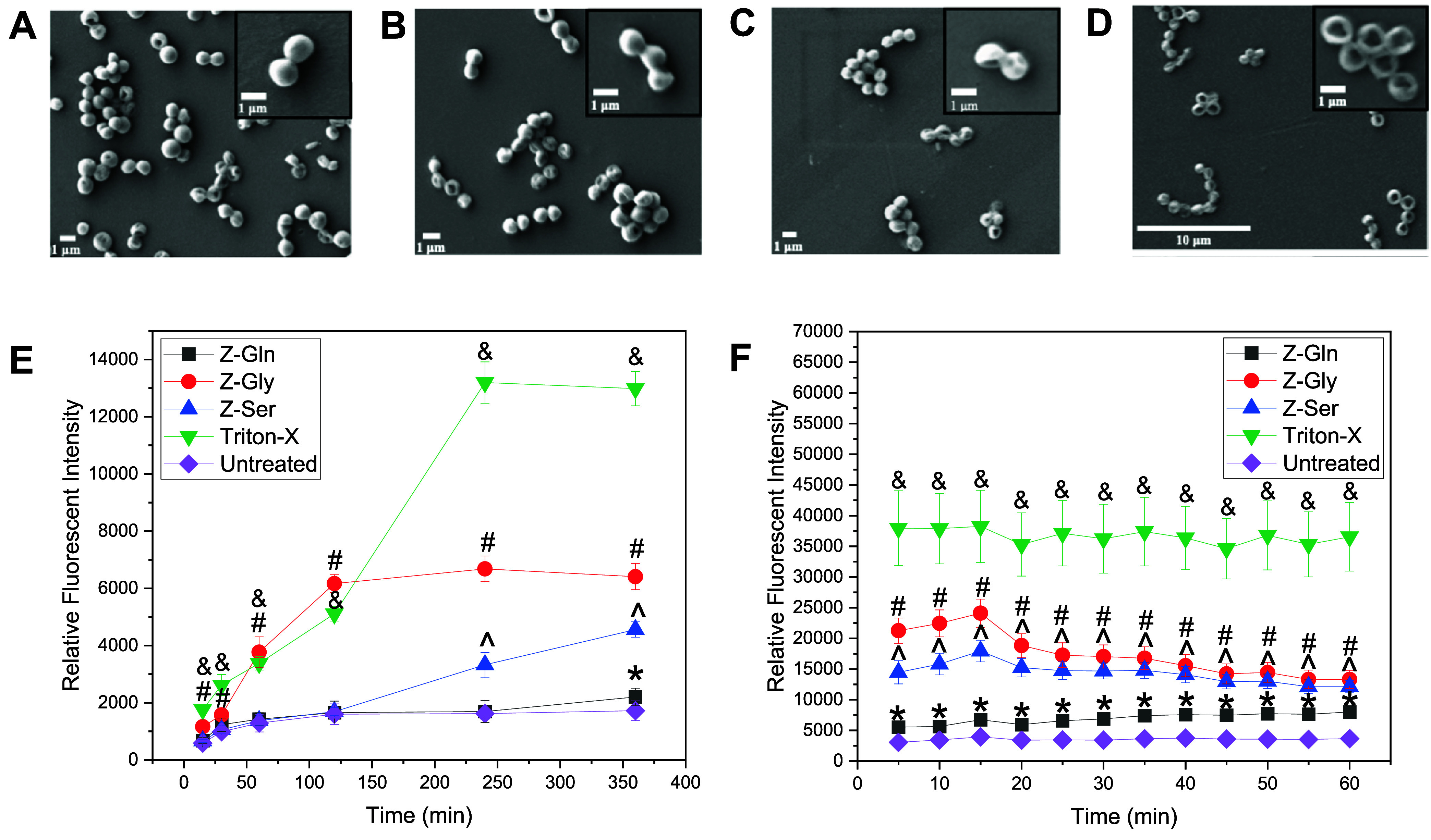
Effects of Z-amino acids on *S. aureus* (MSSA) morphology, membrane permeability, and membrane potential. Representative images showing *S. aureus* morphology under different treatment conditions: (A) Untreated control, and treated with (B) l-Gln, (C) 0.5× MIC Z-Gln, and (D) 1× MIC Z-Gln. (E) Permeabilization and (F) depolarization of *S. aureus* membranes treated with Z-amino acids at 1× MIC. 0.1% Triton-X was used as a positive control. * < 0.05 (Z-Gln), ^#^ < 0.05 (Z-Gly), ^∧^ < 0.05 (Z-Ser), and ^&^ < 0.05 (Triton-X) compared to the untreated control. Data are represented as the mean ± standard deviation with *n* = 6 replicates.

### 3.4 Cell Viability

The toxicity of Z-amino acids on mammalian cells, including osteoblasts and BEAS-2B cells was determined. Exposure times were selected based on antimicrobial efficacy: 1 h was chosen as Z-Gly achieved over 90% bacterial inhibition at this time point; 4 h corresponded to >90% inhibition of MRSA in Z-Gln and Z-Gly; and 6 h was evaluated since this time point inhibited >90% of MSSA for Z-Gln and Z-Gly (Figure 2). Osteoblast viability seemed to be dependent on the Z-amino acid concentration (Figure 6A). Z-Gln resulted in high osteoblast viability (>70%) at 0.5×, 1×, and 2× MICs, but had significantly lower viability (∼12%) at 4× MIC. Z-Gly had high osteoblast viability (∼95%) at 0.5× MIC, which decreased significantly to below 50% at 1× MIC and further declined to ∼12% at 2× and 4× MIC. Z-Ser showed high osteoblast viability at 0.5× and 1× MIC, but had significantly lower viability at 2× and 4× MIC (Figure 6A). At 1× MIC, Z-Gln had the highest viability (83%), followed by Z-Ser (77%), and Z-Gly had the lowest viability (46%), which was significantly lower compared to the control and other Z-amino acids (i.e., Z-Gln and Z-Ser). At 2× MIC, Z-Gln had significantly higher viability (81%) compared to both Z-Gly and Z-Ser, and Z-Ser had significantly higher viability than Z-Gly. At 4× MIC, the cell viability reduced to about 12% for all of the Z-amino acid treatments, with no significant differences observed between the treatments (Figure 6A).

**Figure 6:**
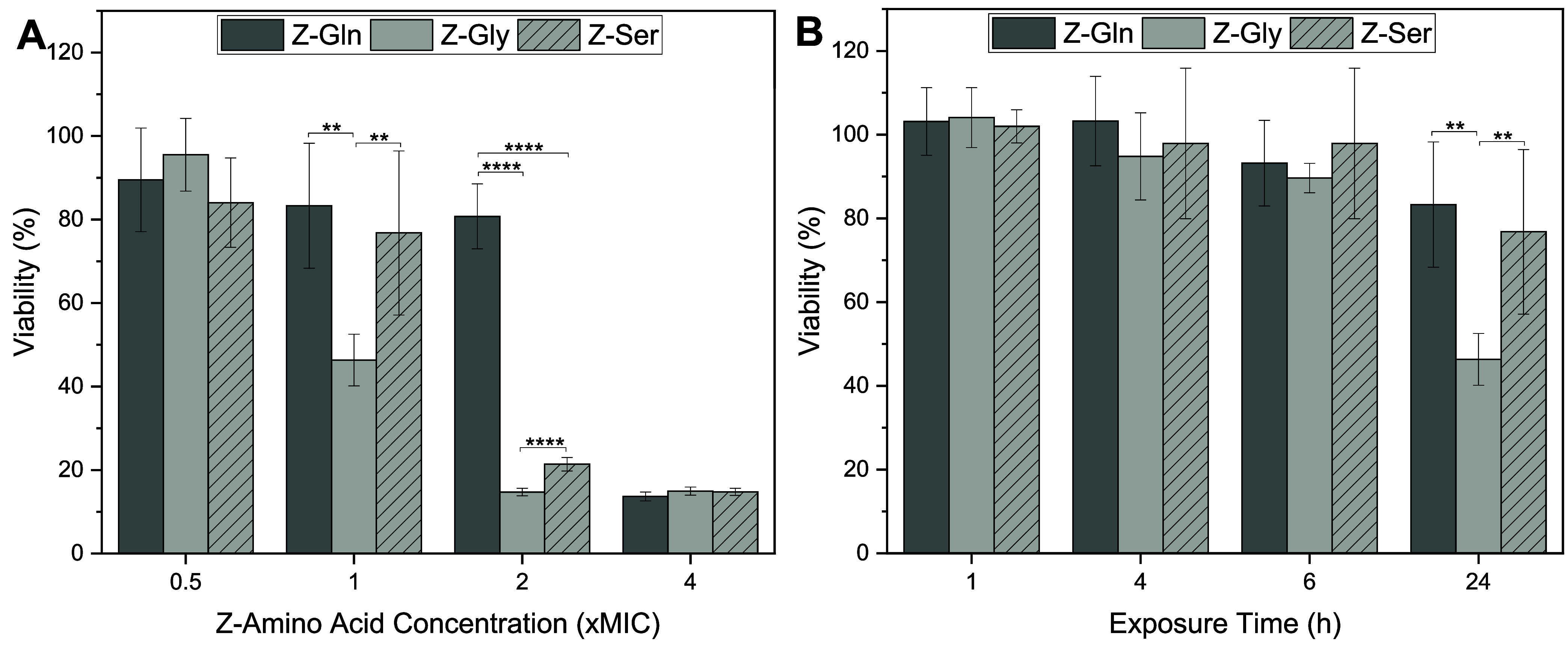
Effects of Z-amino acids on viability of osteoblast cells (A) treated for 24 h with increasing concentrations of Z-amino acids including levels relevant to bacterial inhibition and killing and (B) treated for various exposure times at 1× MIC of each Z-amino acid. ** < 0.01 and **** < 0.0001. Different letters indicate significant differences between Z-amino acid concentrations or exposure times: (a, b) for Z-Gln, (m, n, o) for Z-Gly, and (w, x, y) for Z-Ser (*p* < 0.05). Bars sharing a same letter are not statistically different, and bars labeled with multiple letters (e.g., “a,b” or “w,x”) are not significantly different from either group. Data are represented as the mean ± standard deviation with *n* = 6 replicates.

Overall, osteoblast viability decreased with increasing treatment time at 1× MIC (Figure 6B). Z-Gln maintained high osteoblast viability (>70%) within 24 h. Z-Gly had high osteoblast viability (>90%) at 1 and 4 h, with a significant reduction at 6 h compared to earlier time points, although viability remained above 90%. However, the viability significantly reduced after exposure to Z-Gly at 24 h. Osteoblast viability remained above 70% after a 24 h exposure to Z-Ser, with a significant decrease in viability observed at 24 h compared to earlier time points. After 1, 4, and 6 h exposures, all Z-amino acids had cell viabilities of 90% or higher with no significant differences observed (Figure 6B).

The effects of Z-amino acid treatment on the viability of BEAS-2B cells were also evaluated and compared with those of osteoblast viability. BEAS-2B cells had high viability at 0.5×, 1×, and 2× MIC and had significantly lower viability at 4× MIC (Figure 7A). At 0.5×, 1×, and 2× MIC after a 24 h exposure, there were no statistically significant differences in viability observed between osteoblast and BEAS-2B cells (Figure 7A). However, at 4× MIC, BEAS-2B cells had significantly higher viability compared to osteoblasts. When exposed to 1× MIC Z-Gln for various time points, BEAS-2B cells had ∼80% viability at 1 and 4 h treatment, and cell viability was relatively higher at 6 and 24 h (Figure 7B). Compared to osteoblast viability, BEAS-2B had significantly lower viability at 1 and 4 h but significantly higher viability at 6 h; no significant differences were observed at 24 h (Figure 7B).

**Figure 7:**
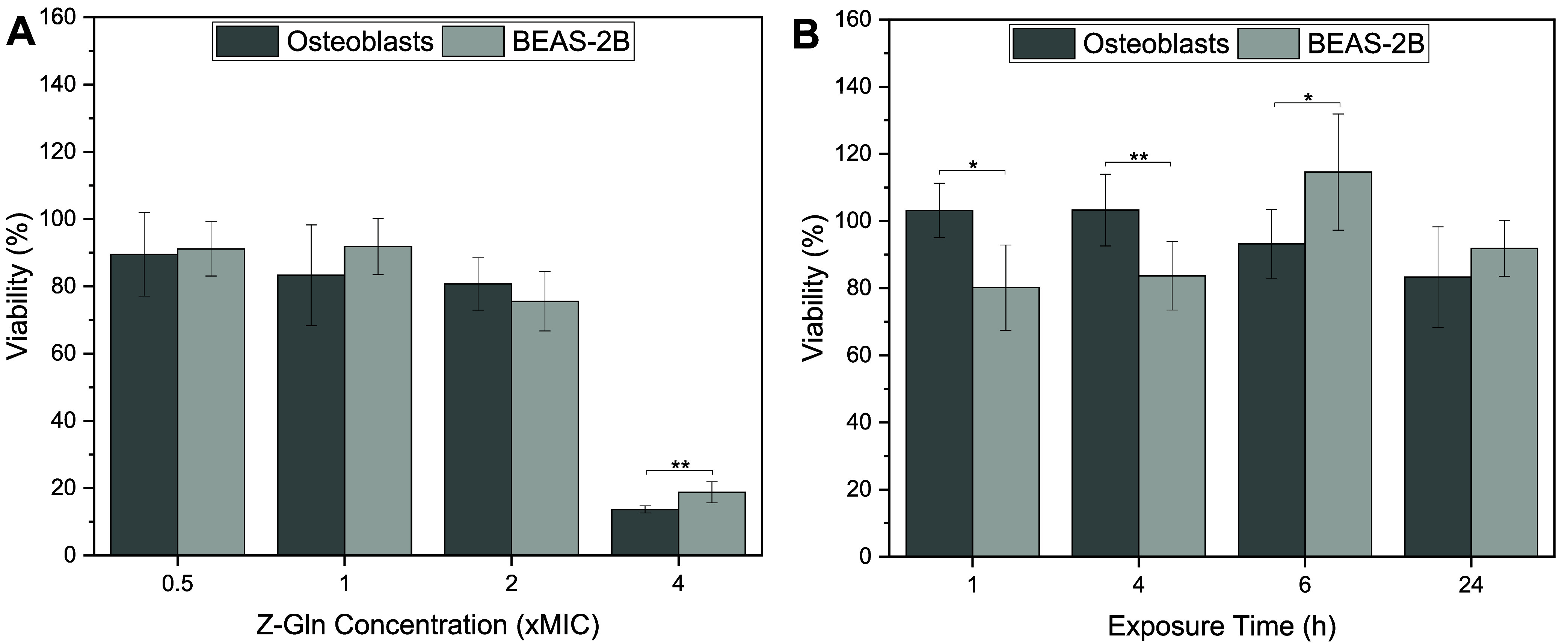
(A) Cell viability of osteoblast and BEAS-2B cells at various concentrations at 24 h of exposure time to Z-Gln. (B) Cell viability of osteoblast and BEAS-2B cells at 1× MIC of Z-Gln for various time points. * < 0.05 and ** < 0.01. Data are represented as the mean ± standard deviation with *n* = 6 replicates.

## 4 Discussion

The global threat of AMR has escalated over the past decade, with biofilm formation being a critical factor in creating resistance to antimicrobial agents.^51,52^ In orthopedic surgeries, approximately 5% result in implant-associated infections, with biofilms being the primary cause of implant failure.^53,54^ Therefore, identifying novel antimicrobial agents that can target both planktonic bacteria and inhibit biofilm formation is essential to overcoming current treatment limitations.

In this study, we evaluated the antimicrobial and antibiofilm properties of Z-amino acids, compounds typically used in peptide synthesis, to determine their potential as independent antimicrobial agents and understand their antimicrobial mechanisms. Among the three Z-amino acids tested, Z-Gly was the most potent against MRSA and MSSA, with the fastest killing kinetics (Figure 2), inhibiting biofilm formation at sub-MIC concentrations (Figure 3), and dispersing biofilms at 1× MIC or lower depending on the strain (Figure 4). Z-Gln and Z-Ser had similar potency, though Z-Ser was more effective against MSSA (Table 1 and Figure 1); both exhibited relatively slower bacterial killing kinetics against MSSA (Figure 2). Beyond planktonic activity, Z-Gln and Z-Ser had lower potency compared to Z-Gly for biofilm inhibition (Figure 3) and biofilm dispersion (Figure 4). Interestingly, sublethal concentrations (e.g., 0.5× or 1× MIC) of Z-Gln and Z-Ser could enhance biofilm formation under certain conditions (Figures 3 and 4), likely due to stress-induced responses, a phenomenon consistent with the literature.^55–57^ As reported in previous studies,^27,58^ our results confirmed that none of the L-amino acids exhibited antimicrobial or antibiofilm properties (Figures 1 and 3), which indicates that the Z-group is essential for the antimicrobial activities observed in the Z-amino acids. Our findings support membrane disruption as a key antimicrobial mechanism of action for Z-amino acids. Z-amino acids induced membrane permeability and depolarization (Figure 5E,F), which was confirmed by the physical disruption of the bacterial membranes observed via SEM (Figure 5D).

When both the antimicrobial efficacy and cytotoxicity were taken into consideration, Z-Gln was the most promising candidate. Table 2 summarizes the effective concentrations of Z-amino acids with regard to planktonic bacteria, biofilm, and osteoblast viability. One can see that Z-Gln at 2× MIC is effective against both MSSA and MRSA while achieving high mammalian cell viability. Although Z-Gly was the most potent against planktonic bacteria and biofilms, it had the highest toxicity at MIC concentrations and after prolonged exposures. Since Z-Gly could inhibit biofilms at sub-MIC levels, which have lower toxicity, it could be suitable for rapid bacterial killing with controlled exposure (Figures 1, 3, and 6). Z-Ser exhibited moderate antimicrobial activity and moderate toxicity, with limited use at higher concentrations required for biofilm dispersal (Figures 1, 4, and 6). In contrast, Z-Gln maintained >70% viability in osteoblasts and BEAS-2B cells at 1× MIC and 2× MIC (Figures 6 and 7), with antibiofilm activity and bactericidal effects at nontoxic concentrations (Figures 1, 4, and 5). These properties make Z-Gln essential for applications requiring longer exposure times. Given the differences in activity and toxicity, the amino acid structure with its amino group protected by the Z-group appears to influence both the antimicrobial activity and cytotoxicity. However, the exact mechanism by which these structural differences affect the efficacy is unclear and requires further investigation.

**Table 2:** Antimicrobial and Cytocompatibility Activities of Z-Amino Acids against MSSA and MRSA

bacteria	MSSA			MRSA		
Z-amino acid	Z-Gln	Z-Gly	Z-Ser	Z-Gln	Z-Gly	Z-Ser
planktonic^a^	≥1× MIC	≥1× MIC	≥1× MIC	≥1× MIC	≥1× MIC	≥1× MIC
biofilm inhibition^b^	≥1× MIC	≥0.5× MIC	≥1× MIC	≥1× MIC	≥1× MIC	≥1× MIC
biofilm dispersal^c^	≥2× MIC	≥1× MIC	≥2× MIC	≥2× MIC	≥0.5× MIC	≥1× MIC
viability^d^	≤2× MIC	≤0.5× MIC	≤1× MIC	≤2× MIC	≤0.5× MIC	≤1× MIC
recommended concentration	2× MIC	—	—	2× MIC	—	1× MIC

a Concentration required to inhibit ≥ 99.9% of bacteria.

b Concentration required to prevent ≥ 50% of biofilm formation.

c Concentration required to reduce ≥ 50% mature biofilm biomass.

d Concentration at which ≥ 70% of osteoblasts remain viable.

Studies with other protected amino acids (such as Fmoc-protected amino acids) or D-amino acids similarly showed either antimicrobial and/or antibiofilm properties. For instance, Fmoc-Phe presented varying antimicrobial activities in hydrogels with MICs of 2 μg/mL^14^ or <500 μM.^22^ Fmoc-Phe also exhibited antimicrobial activity in solution phases with an MIC of 0.46 mM and an MBC of 1.2 mM.^18^ Other reports used a combination of Fmoc-amino acids, such as coassembled hydrogels of Fmoc-Phe and Fmoc-Leu, which inhibited 95% of *S. aureus* proliferation after a 20 h incubation.^20^ Fmoc-amino acids were also shown to inhibit and eradicate biofilm formation in *S. aureus*.^23^ Similarly, D-amino acids had antibiofilm properties with dispersion requiring higher concentrations at 10 mM, whereas inhibition required <100 μM.^24^ However, other reports of D-amino acids indicated more potent biofilm inhibition, with d-Tyr exhibiting biofilm inhibition properties as low as 3 μM^27^ and dispersing biofilms at concentrations >5 mM.^30^ Although D-amino acids were shown to inhibit and disperse biofilms at concentrations lower than those of the Z-amino acids studied here, they did not exhibit significant antimicrobial activity against planktonic bacteria. In comparison, Z-amino acids showed comparable or faster killing kinetics and effective biofilm disruption, though their MICs are higher than what was observed in Fmoc-Phe. While Z-amino acids exhibited promising antimicrobial and antibiofilm activity, their MIC values are over 1000-fold higher than those of conventional antibiotics. For instance, vancomycin inhibits *S. aureus* at MIC values <2 μg/mL for susceptible strains,^59,60^ whereas Z-amino acids required 4 mg/mL to be effective. Despite their higher MICs compared to conventional antibiotics, Z-amino acids had biofilm inhibition at concentrations that remained low or limited cytotoxicity toward mammalian cells, with treated mammalian cells retaining over 70% viability for Z-Gln and Z-Ser.

The findings highlight the potential of a class of modified amino acids, specifically, Z-amino acids, as antimicrobial agents with activity against planktonic bacteria and biofilms. AMPs with higher hydrophobicity have been reported to present enhanced permeabilization in bacterial membranes.^61^ This concept is relevant to our findings as the hydrophobic Z-group may enhance interactions with bacterial membranes. In our study, Z-amino acids caused membrane disruption including depolarization and permeability. This effect is also observed in Fmoc-protected amino acids, which have been shown to exhibit antimicrobial and antibiofilm activities, primarily due to surfactant-like characteristics.^23^ Although Z-amino acids share similarities to Fmoc-amino acids, they are smaller and simpler molecules while maintaining sufficient hydrophobicity to interact with bacterial membranes without requiring bulkier aromatic groups. Z-amino acids exhibit membrane disruption mechanisms and relatively low toxicity at therapeutically relevant concentrations, making them promising candidates for antimicrobial agents alone or in combination with conventional antibiotics. These features highlight the potential of Z-amino acids to be used as antimicrobial candidates, especially to treat biofilm-associated infections commonly associated with antibiotic-resistant bacteria.

This work is limited by focusing on one type of bacteria. Although biofilms typically contain multiple types of bacteria, we selected *S. aureus* primarily due to its prevalence in healthcare-associated infections.^62^ Additional studies should evaluate whether Z-amino acids exhibit broad-spectrum activity by testing against a range of Gram-positive and Gram-negative bacteria. In the future, combination strategies should be explored to determine if there is any synergy between antibiotics and Z-amino acids, which may enhance efficacy, especially against antibiotic-resistant bacteria. Although Z-amino acids demonstrate promising *in vitro* data on the antimicrobial and antibiofilm properties, *in vivo* studies are still needed to evaluate their therapeutic applications. Given their individual antimicrobial activity, Z-amino acids may have the potential to enhance the activity of AMPs when incorporated into their sequences, making this approach promising for improvement of peptide-based therapeutics.^63^

## 5 Conclusions

Z-amino acids exhibited antimicrobial activity and antibiofilm activity with minimal toxicity toward mammalian cells at effective therapeutic concentrations. Although Z-Gln was not the most potent Z-amino acid, it had the most favorable profile against both MSSA and MRSA, maintaining over 70% viability at 1× and 2× MIC, concentrations effective for both planktonic and biofilm activity. Z-Ser also maintained >70% viability at concentrations showing antimicrobial and antibiofilm activity against MRSA (≤1× MIC), but not MSSA (Table 2). Z-Gly had the fastest kinetics, effectively killing *S. aureus* within 1 h at 1× MIC, which had no change in osteoblast viability compared to the control. However, the osteoblast viability significantly decreased after 24 h of exposure, indicating the importance of limited exposure in therapeutic applications. Interestingly, Z-Gln (e.g., 0.125× MIC) and Z-Gly (≤0.5× MIC) stimulated planktonic *S. aureus* growth at lower concentrations. Z-Gln and Z-Ser also increased biofilm biomass compared to the control at lower concentrations (e.g., 0.5× MIC). Overall, at 1× or 2× MIC, Z-amino acids exhibited rapid antimicrobial activity toward *S. aureus*, including fast depolarization and increased membrane permeability, effectively killing bacteria within 6 h. Moreover, Z-amino acids showed the ability to inhibit and disperse biofilm growth, highlighting their use as dual-functional agents in addressing challenges associated with AMR. In addition, we confirmed that L-amino acids do not present antimicrobial or antibiofilm properties, and in some cases may even stimulate bacterial growth. In future studies, the antimicrobial and antibiofilm activity of Z-amino acids against other types of bacteria or their activity in combination with antibiotics will be considered *in vitro* and *in vivo*.

## Abbreviations

AMPs: antimicrobial peptides
AMR: antimicrobial resistance
Boc: tert-butoxycarbonyl
CFU/mL: colony-forming units/mL
Fmoc: fluorenylmethyloxycarbonyl
l-Gln: l-glutamine
l-Gly: l-glycine
l-Ser: l-serine
MBC: minimum bactericidal concentration
MIC: minimum inhibitory concentration
MRSA: Methicillin-resistant *Staphylococcus aureus*
MSSA: Methicillin-susceptible *Staphylococcus aureus*
S. aureus: *Staphylococcus aureus*
Z-amino acids: benzyloxycarbonyl-protected amino acids
Z-Gln: Z-glutamine
Z-Gly: Z-glycine
Z-Ser: Z-ser
